# Toward sustainable environmental quality: Identifying priority research questions for Latin America

**DOI:** 10.1002/ieam.2023

**Published:** 2018-02-22

**Authors:** Tatiana Heid Furley, Julie Brodeur, Helena C Silva de Assis, Pedro Carriquiriborde, Katia R Chagas, Jone Corrales, Marina Denadai, Julio Fuchs, Renata Mascarenhas, Karina SB Miglioranza, Diana Margarita Miguez Caramés, José Maria Navas, Dayanthi Nugegoda, Estela Planes, Ignacio Alejandro Rodriguez‐Jorquera, Martha Orozco‐Medina, Alistair BA Boxall, Murray A Rudd, Bryan W Brooks

**Affiliations:** ^1^ Aplysia Environmental Consulting Vitória Brazil; ^2^ Instituto de Recursos Biológicos, Centro de Investigaciones de Recursos Naturales (CIRN) Instituto Nacional de Tecnología Agropecuaria (INTA) Buenos Aires Argentina; ^3^ Pharmacology Department Federal University of Parana Curitiba Brazil; ^4^ CIMA Universidad Nacional de la Plata – CONICET Buenos Aires Argentina; ^5^ Department of Environmental Science Baylor University Waco Texas USA; ^6^ Department of Chemistry Federal University of São Carlos São Carlos Brazil; ^7^ IQUIBICEN‐CONICET Universidad de Buenos Aires Buenos Aires Argentina; ^8^ Universidade Estadual de Feira de Santana Feira de Santana Brazil; ^9^ Laboratorio Ecotoxicología y Contaminación Ambiental, IIMyC, CONICET‐UNMDP Argentina; ^10^ Laboratorio Tecnológico del Uruguay (LATU) Montevideo Uruguay; ^11^ INIA, Department of Environment Madrid Spain; ^12^ RMIT University Melbourne Australia; ^13^ National Institute of Industrial Technology Chemistry Center Buenos Aires Argentina; ^14^ Centro de Humedales Río Cruces Universidad Austral de Chile Valdivia Chile; ^15^ University of Guadalajara Guadalajara Mexico; ^16^ Environment Department University of York York United Kingdom; ^17^ Department of Environmental Sciences Emory University Atlanta Georgia USA

**Keywords:** Sustainable development goals, Chemicals and waste, Urbanization, Environmental stressors, Environmental quality research needs

## Abstract

The Global Horizon Scanning Project (GHSP) is an innovative initiative that aims to identify important global environmental quality research needs. Here we report 20 key research questions from Latin America (LA). Members of the Society of Environmental Toxicology and Chemistry (SETAC) LA and other scientists from LA were asked to submit research questions that would represent priority needs to address in the region. One hundred questions were received, then partitioned among categories, examined, and some rearranged during a workshop in Buenos Aires, Argentina. Twenty priority research questions were subsequently identified. These research questions included developing, improving, and harmonizing across LA countries methods for 1) identifying contaminants and degradation products in complex matrices (including biota); 2) advancing prediction of contaminant risks and effects in ecosystems, addressing lab‐to‐field extrapolation challenges, and understanding complexities of multiple stressors (including chemicals and climate change); and 3) improving management and regulatory tools toward achieving sustainable development. Whereas environmental contaminants frequently identified in these key questions were pesticides, pharmaceuticals, endocrine disruptors or modulators, plastics, and nanomaterials, commonly identified environmental challenges were related to agriculture, urban effluents, solid wastes, pulp and paper mills, and natural extraction activities. Several interesting research topics included assessing and preventing pollution impacts on conservation protected areas, integrating environment and health assessments, and developing strategies for identification, substitution, and design of less hazardous chemicals (e.g., green chemistry). Finally, a recurrent research need included developing an understanding of differential sensitivity of regional species and ecosystems to environmental contaminants and other stressors. Addressing these critical questions will support development of long‐term strategic research efforts to advance more sustainable environmental quality and protect public health and the environment in LA. *Integr Environ Assess Manag* 2018;14:344–357. © 2018 The Authors. *Integrated Environmental Assessment and Management* published by Wiley Periodicals, Inc. on behalf of Society of Environmental Toxicology & Chemistry (SETAC)

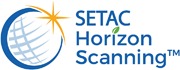

## Key Points

- The Global Horizon Scanning Project aims to identify timely research needs around the world that are necessary to achieve more sustainable environmental quality.
- Twenty priority research questions for Latin America were identified, following input from multidisciplinary scientists and engineers from academia, government, and business and a synthesis workshop.
- Addressing these critical questions will support development of a long‐term strategic research effort to advance more sustainable environmental quality and protect public health and the environment in Latin America.

## INTRODUCTION

What research is needed to achieve more sustainable environmental quality? Global megatrends such as demographic transitions, urbanization, and the food–energy–water nexus continue to transform international relations, while stressing critical resources and affecting public health and the environment. Environmental challenges are pervasive and inherently vary within and among geographic regions. To address these challenges, nongovernmental, governmental, business, and academic entities routinely identify needs to advance strategic goals. One well‐known example is the United Nation's 2030 goals for sustainable development (http://www.un.org/sustainabledevelopment/sustainable-development-goals). The global goals specifically include 17 sustainable development goals and 169 targets, which aim to extend beyond and complete the previous Millennium Development Goals from 2000 (http://www.unmillenniumproject.org/goals). Many of these sustainable development goals depend on implementation of environmental management decisions. Identification of specific research programs to support environmental management goals often occurs within organizations in response to societal needs, and then is modified as priorities and resource availability change through time. Scientists and engineers have thus responded to numerous environmental issues, yet interdisciplinary efforts to prospectively identify specific research necessary to address environmental quality challenges have not occurred at the global level (Brooks et al. 2013).

Because credible scientific answers to policy‐relevant issues remain critically important, the United Nations employs integrated environmental assessments to support evidence‐based environmental decisions. These assessments are routinely included as the foundation for United Nations’ Global Environment Outlook (GEO) reports (http://web.UNEP.org/geo/). Interestingly, a category related to chemicals and waste was included in GEO 5, which was published in 2012, for the first time. Ongoing efforts include development of GEO 6. Of particular importance to Latin America (LA), GEO reports target specific regions with the most recent report for LA and the Caribbean (LAC) published in 2016 (http://web.UNEP.org/geo/). The most pressing issues for LAC included threats to biodiversity, habitat degradation, pollution, climate change susceptibility, and unsustainable patterns of production and consumption (http://web.UNEP.org/geo/). Clearly these topics deserve immediate attention, yet no attempt has been made to identify key environmental research questions associated with these pressing issues for LA. Herein, horizon scanning, including “key questions” methods (Sutherland et al. 2009; Sutherland and Woodroof 2009), presents a useful approach to identify credible and tractable, and potentially more legitimate, research questions for which timely answers are needed, particularly given financial constraints (Rudd et al. 2014). Thus, the Global Horizon Scanning Project (GHSP) was initiated to identify multidisciplinary scientific research needs that, if answered, would achieve more sustainable environmental quality around the world (Brooks et al. 2013). In the present paper, we report findings from a horizon scanning effort aimed to identify key environmental quality research questions for LA.

## METHODS

In the present study, we followed methods previously reported by our research team (Boxall et al. 2012; Rudd et al. 2014), which included distributing surveys to environmental scientists and engineers from the academic, business, and government sectors in LA. These Internet‐based surveys were sent to recent meeting attendees and members of the Society of Environmental Toxicology and Chemistry's (SETAC) LA geographic unit, the Brazilian Society of Ecotoxicology, and other scientists and engineers in LA. Key questions were requested to address important gaps in knowledge, be answerable through a realistic research design, have factual answers that do not depend on value judgments, cover a spatial and temporal scale that could realistically be addressed by a research team (e.g., €10 million over 5 y), not be answerable by “it all depends” or “yes” or “no,” and if a question was related to impact and interventions, it should have contained a subject, an intervention, and a measurable outcome (Boxall et al. 2012; Rudd et al. 2014). These questions were intended to be scientific and reflective of technical perspectives from LA, in a global context. All input received from this survey (100 total) is provided as Table S1 in Supplemental Data. The process, therefore, was intentionally inclusive, bottom–up, multidisciplinary, multisector, and transparent.

**Figure 1:**
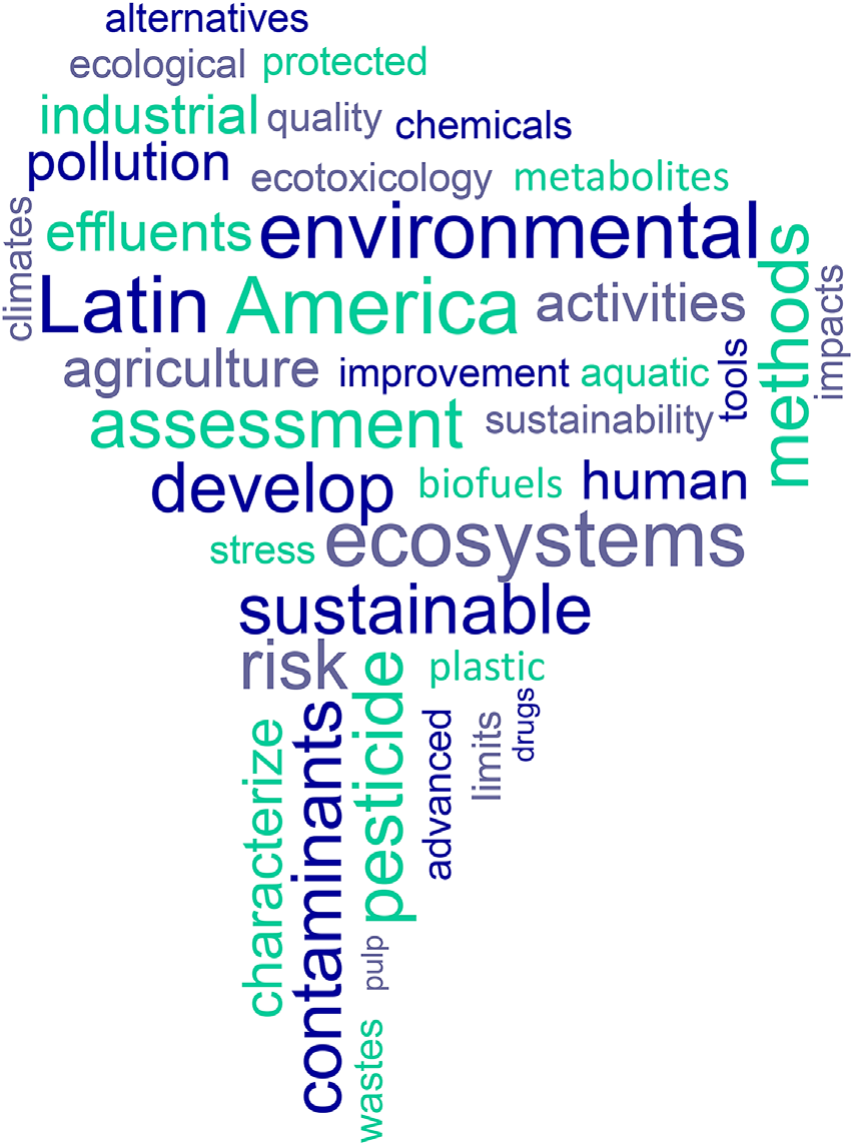
Word cloud of key terms from 20 priority environmental quality research questions for Latin America.

Submissions from LA were partitioned by the steering committee among major themes, including environmental chemistry, ecotoxicology, risk assessment, environmental management and policy, and an integrative category focusing on health, contaminants of emerging concern, and the environment. An additional theme included research questions of specific relevance to LA. These 6 themes were then the subject of breakout discussion groups during a workshop held in Buenos Aires, Argentina, in 2015. Following such initial partitioning among themes, 20 priority research questions were identified, which included some redevelopment of initial questions with similar or complementary content, by interdisciplinary participants from the academic, business, and government sectors following previously reported methods (Sutherland et al. 2011; Boxall et al. 2012). We critically examine these key research questions in the following sections (Figure 1).

## ENVIRONMENTAL CHEMISTRY

### What are the levels of pollution by plastic waste and microplastics, and are toxic compounds adsorbed on the surface of the plastic?

Mass production of plastics began in the 1940s but concerns about the potential environmental impacts of large plastic debris, “macroplastics,” were identified in the early 1970s (Carpenter and Smith Jr 1972). However, the risk of small or microscopic plastic fragments (<5 mm) as pollutants was not considered until the start of the new century (Andrady 2011; Cole et al. 2011; Duis and Coors 2016). Though a few recent studies on the presence of microplastics in marine environments are available from Brazil, Colombia, Uruguay, and Chile (Costa et al. 2010; Acosta‐Coley and Olivero‐Verbel 2015; Lozoyaa et al. 2015; Rech et al. 2015), studies in LA are relatively limited. Further, a large knowledge gap exists on the ecotoxicological effects of microplastics (Cole et al. 2011). In addition to physical injuries that plastic waste and microplastics can cause to wildlife through abrasions and blockages, aquatic and terrestrial organisms may also be negatively affected by plastics and microplastics through exposure to chemicals released from these materials. Moreover, due to their large surface area to volume ratio, microplastics can become heavily contaminated with waterborne hydrophobic persistent organic pollutants (POPs) (Wardrop et al. 2016) and other contaminants, and thus pose a possible route of chemical exposure to aquatic organisms, which may result in bioaccumulation and biomagnification through the food chain. Wright et al. (2013) identified the need for studies that focus on evaluating the capacity for microplastics and their associated contaminants to be transported along marine food webs via trophic interactions as well as an estimation of population‐ and ecosystem‐level impacts. To our knowledge, no studies in LA have examined the bioaccumulation of substances released from plastics and their associated contaminants across trophic levels.

### Considering the bioaccumulative potential of some active pharmaceutical compounds, metabolites, and their mixtures in aquatic organisms, how can we assess the implications in a long‐term perspective for human health and the environment? How can we insert these limits in environmental legislation?

The risk of exposure to pharmaceutical mixtures can vary in different geographic regions due to population demographics, cultural practices, environmental and climatic characteristics, dilution potential of receiving environments, and infrastructure related to wastewater and drinking water treatment (Boxall et al. 2012; Kookana et al. 2014). In most LA cities, raw or poorly treated sewage is discharged to surface waters. In addition, development of shantytowns around big cities with no sewage connectivity and treatment contributes to uncontrolled sewage discharge to streams, rivers, and lakes across large areas of LA (Kookana et al. 2014). Some studies recently conducted in Argentina and Brazil have shown that pharmaceuticals are present in wastewater effluents and receiving waters (Elorriaga et al. 2013a, 2013b; Thomas et al. 2014; Valdés et al. 2014, 2015; Campanha et al. 2015). However, almost nothing is known about the bioaccumulation and biomagnification potential of pharmaceuticals (including metabolites and mixtures) in food webs of the highly diverse aquatic and terrestrial ecosystems of LA. Understanding bioaccumulation of pharmaceuticals in wildlife was recently identified as an important research need (Boxall et al. 2012). Other important research needs (Boxall et al. 2012) include understanding the fate and effects of pharmaceutical metabolites and degradates and their mixtures. Because most pharmaceuticals are excreted and released into the environment as biologically active metabolites, studies are needed to identify and better understand these metabolites, and the formation of other transformation products, which could pose lesser or greater risks in the environment than the parent compound. Long‐term human health and ecological risks associated with pharmaceutical mixtures are not understood, which requires development of and access to instrumentation to monitor these compounds in food and drinking waters. Clearly, future research studies are needed in this area, particularly in LA.

Environmental legislation should be strongly grounded in the science. Similar to the development of legislation regarding other contaminants, it will be necessary to develop systems that ensure the incorporation of environmental chemistry and ecotoxicological information and criteria into legislation regarding contaminants of emerging concern, including pharmaceuticals. For example, minimum selective concentrations for the development of antibiotic resistance (Bengtsson‐Palme and Larsson 2015) and therapeutic hazard values (Brooks 2014) have been proposed for antibiotics and other pharmaceuticals, respectively, but these diagnostic screening values have not been examined in LA. Consequently, it will be important to expand scientific research studies that aim to better understand responses to low concentrations of pharmaceuticals and their metabolites and mixtures in the environment. Another challenge in LA will be to build professional capacity and to set up laboratories able to analyze low concentrations of pharmaceuticals and other contaminants in water, soils and sediments, and biota (plant and animal tissues).

### How can we better quantify contaminants (e.g., pesticides), metabolites, and degradation products in the field, and develop more robust methods for analytical determination in plant and animal tissues?

Identifying how naturally occurring and anthropogenic chemicals elicit adverse outcomes to humans and ecosystems remains a pressing research need. Determination of chemical residues in contaminated food, air, and water critically supports an understanding of the magnitude, frequency, and duration of environmental exposure and potential risks. Fortunately, technological advances provide new opportunities to determine the spatial and temporal patterns of parent compounds, metabolites, and degradation products in various environmental matrices, including plant and animal tissues, including human biomonitoring. In fact, when coordinated environmental specimen–banking programs are coupled with advanced analytical methodologies, such as high‐resolution, nontarget‐directed chemical analysis and innovative contaminant monitoring systems (e.g., mobile phones, passive samplers, microsensing networks), it becomes possible to more rapidly identify risks and prioritize locations that require public health and environmental interventions. For example, the US Centers for Disease Control and Prevention National Health and Nutrition Examination Survey (https://www.cdc.gov/nchs/nhanes/) and the German Environment Agency's Environmental Specimens Bank (http://www.umweltbundesamt.de/en/topics/health/assessing-environmentally-related-health-risks/environmental-specimen-bank) provide useful models that could be expanded to other regions.

Routine access to advanced analytical instrumentation and other innovative technologies remains elusive in many LA countries, where resources are commonly focused to face social issues (e.g., poverty, health) more than to tackle environmental problems. Building or consolidating a few strategically distributed, high‐complexity research centers devoted to providing services to large regional areas (even between countries) with shared environmental problems would help to optimize resources and capabilities. Production of chemicals for industrial, agricultural, and personal use is increasing with development and population growth. Moreover, in the megacities of many developing countries, access to and concentration of chemical use is occurring faster than public health and environmental management systems can be effectively implemented (Corrales et al. 2015; Kristofco and Brooks 2017), which presents emerging challenges to water and food security. Therefore, research is needed to develop more rapid, inexpensive, and robust methods for analytical determinations of environmental contaminants, including chemicals of emerging concern, difficult to measure substances, metabolites, and degradation products, in diverse matrices. For example, portable devices, real‐time monitoring, and citizen science approaches could be advanced to identify areas of concern, which can be subjected to more advanced analyses. Further, development of coordinated environmental specimens–banking networks with innovative diagnostic tools, such as passive sampler networks (Lohmann et al. 2017), is needed to advance global chemical monitoring and surveillance, an essential service of environmental public health (https://www.cdc.gov/nceh/ehs/10-essential-services/index.html).

## ECOTOXICOLOGY

### How will climate change influence environmental stress factors (e.g., temperature, pH, salinity), which in turn affect the environmental fate and effects of contaminants?

A strong scientific consensus exists that climate change is occurring and is the result of rising anthropogenic greenhouse gas emissions (IPCC 2014a, 2014b). Shifts in climatic conditions are now affecting wildlife and plant species worldwide through increases in global air and ocean temperatures (IPCC 2013), snow and ice melt (Blunden and Arndt 2014), and enhanced frequency and severity of extreme temperature, drought, and precipitation (Hansen et al. 2012; Huntingford et al. 2013). Of relevance to environmental toxicology and chemistry, potential consequences of climate change include alteration of the environmental distribution and fate of chemicals and subsequent changes in exposure and toxicological response of organisms (Noyes et al. 2009). Indeed, because global contaminants are affected by environmental and climatic factors, the changing climate will undoubtedly affect global processes surrounding the release, volatilization, transport, chemical or physical conversion in the atmosphere, or other media, deposition, and environmental partitioning of contaminants (Sanderson and Goodsite 2015). In addition, environmental changes associated with climate change have the potential to enhance organismal susceptibility to chemical toxicity. Alternatively, chemical exposures themselves may impair the ability of organisms to cope with the changing environmental conditions of the shifting climate (Noyes and Lema 2015). There is growing awareness of the need to anticipate chemical pollution effects in rapidly changing environments and to identify and mitigate adverse outcomes in those human populations and ecosystems most vulnerable (Noyes et al. 2009). Future environmental and ecotoxicology research efforts in LA, and in other regions of the world, will need to focus on the interactions among altered climate, chemical exposure, and species susceptibility to understand multiple stressor influences on human health and the environment (Landis et al. 2014).

### By which means can we evaluate the complexity (i.e., pulse, degradation, mixtures, formulations) of pesticide toxicology in LA ecosystems?

Agriculture in general and grain production in particular provide the economic backbone of many LA countries, and the increased dependence on transgenic crops has resulted in a rapid expansion of pesticide use in the region (Tomei and Upham 2009; Carneiro et al. 2012). Because of the risks associated with pesticide use, countries have established laws and regulations to control the production and use of pesticide products. These regulations are generally based on toxicity data generated through standardized test protocols that normally require continuous exposure of study animals, typically from the northern hemisphere, to a single pesticide. However, differences have been observed among the toxicity of some pesticides we tested with scenarios more closely representative of field conditions in the Pampas compared with standardized laboratory toxicity methods (Carriquiriborde et al. 2007). Additionally, real‐world exposures normally occur as exposure to pulses of mixtures of pesticides, separated by periods of exposure to very low concentrations (Ronco et al. 2008; Dennis et al. 2012; King et al. 2016). This dichotomy between testing methods and real‐world exposures questions the adequacy of current toxicity test protocols for predicting the effects of the pesticides released in the environment. Furthermore, pulsed exposures also render inefficient the classical field monitoring protocols in which a single sample is taken at regular intervals, because these protocols lack the spatial and temporal resolution necessary to capture peaks of exposition (Stehle et al. 2013; Xing et al. 2013). To more accurately evaluate ecological risks of pesticides in LA ecosystems, it is essential to develop new and modern monitoring and toxicity test protocols that better take into account the complexity of pesticide exposures in the field, while more closely coupling results from more advanced and environmentally realistic laboratory and field studies.

### How can we extrapolate the results of regulatory single‐species toxicity tests to LA taxa in different ecosystems, climates, or physicochemical conditions?

Though ecotoxicological research outputs in LA continue to increase from academic institutions, information and knowledge generated by these academic studies that reach regulatory authorities are still very limited. Moreover, ecological hazard and risk assessments, if required, are generally based only on ecotoxicity data from laboratory standardized toxicity assays with organisms from other regions (e.g., the northern hemisphere). In many cases, these data can be inadequate for LA ecosystems due to differences in environmental fate and native species sensitivities (Carriquiriborde et al. 2014). Significant differences in toxicity thresholds appear to exist among temperate and tropical species (Kwok et al. 2007). In addition, the use of indigenous or native species is generally believed to provide more environmental realism and to ensure that sensitive species within ecosystems are being protected (USEPA 1982; Echols et al. 2015). To increase the quality and relevance of ecological risk assessment in LA, it is important to develop and implement LA‐specific regulatory testing protocols using native species selected for both their widespread occurrence and ecological relevance to LA ecosystems (Brodeur and Poliserpi 2017). The development and establishment of regulatory multispecies toxicity tests would also greatly improve risk assessment because these model systems are more realistic and representative of field conditions than are single‐species tests because they evaluate both direct and indirect effects on a population level (Landis et al. 1997; De Laender et al. 2009) in different ecosystems, climates, or physicochemical conditions. To facilitate these activities, the Organisation for Economic Co‐operation and Development (OECD) plays a key role in the development and validation of test guidelines (TGs) for the identification and assessment of the hazard of chemicals. The Agreement for Mutual Acceptance of Data inside OECD countries foresees data acceptance when generated following OECD TGs under good laboratory practices and constitutes an essential tool for sharing information and reducing testing costs. Future participation at the OECD as full or associated members of several LA countries could facilitate international regulatory acceptance of experimental protocols specifically developed with LA species and for LA environmental conditions.

### What new laboratory or field ecotoxicology methods and approaches can be developed to account for ecological and environmental complexity?

The fields of ecotoxicology and ecological risk assessment have made considerable progress over the last 50 y, significantly reducing risks from acute and high‐volume pollution in ecosystems by providing basic information on the large number of chemicals introduced into the environment and by implementing waste management systems and technologies. Nevertheless, the scientific community now recognizes that the risk assessment procedures on which these disciplines normally rely can suffer from a lack of ecological realism and can be simply inadequate to characterize risks to ecosystems and human health following chronic exposures to low concentrations of an increasing number of contaminants (Eggen et al. 2004; Vighi and Villa 2013). For example, some of the important limitations of the field of ecotoxicology include:

1. limited ecological realism of current toxicity testing procedures,
2. difficulty detecting effects of chronic exposures to chemicals at low concentrations,
3. challenges associating mechanistic (e.g., molecular, biochemical) responses to fitness at higher levels of biological organization,
4. difficulty evaluating and predicting effects on populations and communities, and
5. problems associated with assessments of indirect ecological effects (e.g., competition, predation).

Ecotoxicology must continue to advance beyond current limits to achieve its ultimate aim of determining, predicting, and avoiding contaminant effects in real‐world systems across large spatial scales (Beketov and Liess 2012). Herein, although laboratory‐to‐field studies have occurred for decades (Dickson et al. 1992; La Point and Waller 2000), additional conceptual schemes were more recently proposed for reducing uncertainty in laboratory‐to‐field extrapolation (Vignati et al. 2007) and for mechanistically linking responses to chemicals across levels of biological organization to adverse outcomes in individuals and populations (Ankley et al. 2010). For example, a similar field–lab–field iterative process was successfully used for assessing risks of pyrethroids use on soybeans to fish in the Pampas (Carriquiriborde et al. 2007). These and other innovative approaches should be developed and employed more broadly in LA.

## HEALTH, CONTAMINANTS OF EMERGING CONCERN, AND ENVIRONMENT

### What are the impacts of nanomaterials on ecosystems and on human health?

Widespread use of nanoscale materials is causing an increase of environmental concentration. Nanomaterial absorption, distribution, metabolism, and excretion in organisms can appreciably differ from those of other chemicals, due to specific characteristics. There is a marked lack of information about concentrations or amounts of nanomaterials in environmental compartments such as surface waters and soils. Methods for measuring these substances are being developed (von der Kammer et al. 2012). In addition, due to their low solubility and particulate nature, there are enormous difficulties in applying standard tests (e.g., OECD TGs) to nanomaterials in order to identify and assess their hazards, although a large effort is being engaged at the international level for adapting existing assays to be more applicable to nanoparticles (Hund‐Rinke et al. 2016). Due to the behavior of nanoparticles during toxicity studies, it is difficult to compare results from different research groups and determine whether reported toxicity observations are physiologically relevant. Knowledge gaps remain regarding the nature of interaction of nanoparticles within environmental systems, the bioaccumulation and biomagnification of diverse nanoparticles within aquatic and terrestrial organisms, and whether this differentially affects food consumption among species. Nanomaterial accumulation in soil and water bodies through extensive use and production of new technology, spills, runoff, and emissions is of particular importance in LA because legislation dealing specifically with nanomaterials is not currently available.

### Which environmental variables (abiotic and biotic) trigger the production of algal toxins in the environment? Does exposure through trophic levels threaten human health?

The magnitude, frequency, and duration of harmful algal blooms (HABs) in fresh and brackish waters have become a major environmental issue and emerging human health threat at the global scale (Brooks et al. 2016). Harmful algal blooms occur naturally and are caused by interacting factors that vary among algal species (Chorus and Bartram 1999). However, key forcing factors for the development of HABs include climate change and associated droughts, nutrient enrichment, and other modifications resulting from anthropogenic activities such as contaminants from effluent and stormwater discharges, natural resource extraction, agricultural runoff, and salinization. Many HAB‐forming species are invasive and/or opportunistic, and take advantage of altered habitat conditions in developed and developing regions. The HAB impacts are not as predictable as those from conventional chemical contaminants; interactions among multiple factors, both natural and anthropogenic, determine the severity to which an HAB will occur in a specific water body and can affect the magnitude of toxin production. In the case of cyanobacteria HABs, interactions between nutrients (including both N and P) and climate change may exacerbate potential impacts on water resource uses (Paerl and Huisman 2008; Paerl and Paul 2012; Paerl et al. 2016), including drinking water supplies, agriculture, and recreational fishing and swimming. Cyanobacteria HABs result in a variety of water quality problems, such as impairment to recreational uses, reduced aesthetics, lower dissolved O concentrations, taste and odor problems in drinking water, and the production of multiple toxins, often by the same species, which can affect aquatic and terrestrial wildlife and human health. Human exposure to cyanotoxins can occur by ingestion of contaminated fish, shellfish, and drinking water, by inhalation, or by dermal contact. Such HAB events are particularly relevant in tropical and subtropical regions of LA (e.g., Brazil). For example, studies have reported cyanotoxin concentrations in animal tissues (Clemente et al. 2010; Guzmán‐Guillén et al. 2014) and possible transfer over trophic levels (Nogueira et al. 2004).

Unfortunately, a predictive understanding of chemical, physical, and biological influences on production and acute and sublethal consequences of diverse HAB toxins is rarely available, particularly for less studied inland HAB species and the toxins they produce (Brooks et al. 2016). In fact, current capacity to predictively model HAB initiation and termination events and toxin production is extremely limited. However, recent predictive modeling activities, supported through multiyear collaborative effort involving laboratory experiments, in situ studies, and spatially and temporally explicit field monitoring, have successfully predicted bloom formation of an invasive mixotrophic, euryhaline, eurythermal, and relatively understudied HAB species in inland and coastal waters (Grover et al. 2012). These advances, made possible through sustained research, provide a template for developing future modeling efforts to predict other HAB occurrence and severity. In fact, because the causes of HABs have been associated with changes in climate, land use, and water resource management, an improved ability to predict HABs coupled with regional watershed management and planning may enable reduction of adverse outcomes caused by inland HABs. Unfortunately, current water quality models include quite limited cyanobacteria HAB growth and toxin assumptions across environmentally relevant gradients of N:P and salinity. For example, how cyanobacterial HAB toxins production and the risks they pose are influenced by these environmental gradients remains absent in even the most advanced lake and reservoir models. Clearly, developing a predictive understanding of inland HABs and products of associated toxins presents a palpable research need in LA and other regions.

## RISK ASSESSMENT

### How can we characterize individual and combined (e.g., mixtures) risks of diffuse chemical contaminants (e.g., pesticides, other endocrine disrupting chemicals, drugs) related to promoting more sustainable agricultural, urban, and industrial activities?

In LA, the use of pesticides in agriculture represents 52% of world consumption (FAO 2015), and just 15% to 28% of municipal sewage is treated (ONU‐HABITAT 2012). Moreover, the South American crop protection chemicals market has been estimated at US$14.1 billion in 2015 and is projected to reach US$19.6 billion by 2020. The market is also segmented geographically into Brazil, Argentina, and others. Brazil has the largest consumer base in the world, while Argentina follows. Such expansive pesticide use is related to extensive agricultural development in LA. Contaminants related to agricultural, municipal, and industrial activities are collectively introduced to water resources. Some of these contaminants (e.g., pesticides) are evaluated and classified for their environmental risks, but these analyses are done singly, while risks of diffuse pollution and effects of multiple contaminant mixtures are rarely understood (Brodeur et al. 2014, 2016). For example, there is scarce information about the real‐world impact of diffuse agrochemical pollution on agricultural ecosystems and their functions, such as the environmental effects of stressors associated with coffee and sugar cane plantations. It is known that the use of agrochemicals in the field involves pesticide mixtures for which unknown and adverse effects are expected (Miglioranza et al. 2013; Ondarza et al. 2014; Lupi et al. 2016; Silva Barni et al. 2016). Therefore, there is a need to develop tools and implement rules that characterize hazards and risks of the simultaneous use of 2 or more agrochemicals. Similarly, understanding and reducing the potential environmental effects of pesticides in combination with other chemical products used in industry and households represents an important research need, particularly in LA and other developing regions.

### How can environmental risk assessment tools, including alternative methods, be developed and advanced to more sustainably produce, select alternatives, and use chemicals to protect future generations of humans and ecosystems?

Defining levels of sold and used agrochemicals, pharmaceuticals, and industrial chemicals that enter the environment with reliable monitoring surveys to determine biota exposure represents important research needs for LA. Though differential information exists globally for concentrations of various contaminants and their mixtures in the environment, studies examining the fate and effects of these contaminants are rare in LA, and are challenged by limitations of existing risk assessment tools. For example, risks to human health and ecosystems from pesticides and pharmaceuticals, including antibiotics that influence the development of antimicrobial resistant microorganisms, in the LA environment are poorly known. Therefore, understanding adverse chemical effects, selective chemical alternatives, and designing less hazardous substances represent urgent needs, particularly in developing countries where waste management is not consistently implemented.

Development of appropriate alternative methods is becoming a necessity due to ethical and cost issues and often as important tools for obtaining initial insight into the biological activities and hazards associated with thousands of largely unstudied chemicals that are consistently applied and used in the LA region. For example, high‐throughput testing (HTT) efforts are allowing for unprecedented understanding of chemical attributes and associated biological properties with a goal of protection of human health and ecosystems (Schroeder et al. 2016). Advances in other geographical regions (Scholz et al. 2013) could serve as reference and facilitate the implementation of HTT and other alternative methodologies in LA. Similarly, alternative analyses of multiple chemicals for common uses (Dorman et al. 2014) can support chemical substitutions in commercial products that reduce risk to people and the environment (Zimmerman and Anastas 2015). Further, the design of industrial chemicals that maintain function but are inherently less hazardous can protect the environment and human health while providing economic incentives for the design of innovative chemicals and products that allow more sustainable development (DeVito 2016). Herein, significant multidisciplinary research is needed (Coish et al. 2016), but the outcomes promise to be transformational for LA and other regions.

### What are the ecological and health risks and effects characterization methodologies that must necessarily be evaluated to more sustainably manage pulp and paper activities?

In LA, the number of pulp and paper mills has appreciably increased. For example, pulp and paper in LA now represents 14% and 5%, respectively, of the world production (Swedish Forestry Industries Federation 2016). However, little is known about the actual environmental effects of this industrial activity in LA. For example, few initiatives are known, such as those described by Chiang et al. (2011, 2010) and Orrego et al. (2006). Aquatic toxicity from effluent discharges can be influenced by the type of tree (e.g., eucalyptus) plantation to the industrial type of process and by the effluent treatment technologies (Milestone et al. 2012). Studies in temperate countries report potential eutrophication of surface waters and reproductive effects in fish (Fentress et al. 2006; van den Heuvel et al. 2006; Hewitt et al. 2008; Barrett et al. 2010; Martel et al. 2011). Research is needed in LA to ensure selection of chemical and biological endpoints that truly reflect adverse effects in the environment (Hall et al. 2009; Hall and Landis 2009), including the development and broader implementation of robust environmental effects monitoring protocols, such as those employed in Canada (Munkittrick et al. 2005, 2009; Environment Canada 2010).

## ENVIRONMENTAL MANAGEMENT AND POLICY

### What are the progressive goals of continuous improvement of the maximum limits of toxicity and other environmental stress allowed from agriculture (e.g., pesticide runoff, biofuels production), and domestic and industrial effluents, while maintaining ecosystem services?

In LA, only Brazil has federal environmental legislation that includes the assessment of acute and chronic toxicity potential of effluent discharges to watersheds (CONAMA 2011). However, differential monitoring of environmental quality is not sufficient for the maintenance of ecosystem services, and LA has not yet established the continuous improvement targets to reduce the potential for toxic releases. In Brazil, only the Rio Grande do Sul State legislation (CONSEMA 2006) includes progressive targets for reducing the toxicity of industrial and domestic effluents. Conversely, Norway has already set progressive targets for reducing the toxic potential of chemicals used in industrial processes of the oil and gas sector, causing the chemical market to increasingly evolve by using more efficient products that are less toxic in the environment. Efforts should be made to learn from these examples and expand such efforts to other parts of LA.

### Are current environmental regulations (e.g., for effluents, pesticide use) sufficient, and how can they be implemented and enforced in Latin America?

Many aquatic ecosystems in LA are degraded, in particular those in which effluents are discharged to surface waters. Similarly, although new technologies and chemicals are introduced to commerce every year, the efficacy of various treatment infrastructure or other environmental management systems and contaminants releases through effluents and other point and nonpoint sources to the environment are rarely known in LA. Further, regulatory updates occur slowly, similar to other regions. Thus, the use of biological assays for evaluating the effects of environmental contaminant mixtures becomes very important (Grothe et al. 1996; La Point and Waller 2000). In Brazil, several state agencies are using ecotoxicological testing methods as effluent quality risk assessment tools, but in other LA countries, these studies are carried out only in academic settings. For countries that have implemented routine ecotoxicological testing for effluent controls, best local practices can be determined and then serve to launch quality benchmarks from which shorter and longer term targets can be established. Future efforts are needed to advance prospective and retrospective implementation and enforcement of effluent discharges and specialty (e.g., pesticides) and industrial chemical uses. For example, it may be reasonable to consider site‐specific establishment and revision of legal limits of effluent discharges due to the local history of ecotoxicity and environmental status of the basin.

### How can Latin American countries develop, standardize, and harmonize environmental assessment approaches and ecotoxicology methods to advance more sustainable environmental management?

Ecotoxicological tests are standardized in several LA countries and many of these methods are based on international standards (Ramírez‐Romero and Mendoza‐Cantú 2008; Zagatto and Bertoletti 2008; Planes and Fuchs 2015). Work has been done to harmonize ecotoxicological methods in the academic field (Castillo 2004). However, there is no standard protocol common to LA countries with instructions on how to carry out prospective or retrospective ecotoxicological tests for specific chemical contaminants. There are also no common protocols on how to evaluate the environmental effects of effluent discharge in watercourses. Therefore, assessment methodologies vary widely among states within a country and further among LA countries, making it difficult to compare water quality and assess the effectiveness of legal limits for established chemical contaminants. The work of harmonizing the protocols in LA countries will result in the transfer of experiences and knowledge to those countries that are less advanced and will allow better management of effluent quality and watershed integrity. As noted above, the use of already accepted protocols at the OECD level represents a reasonable step forward, considering the increasing number of LA countries that have the status of full or associated members in this organization. At this level, the implementation and use of alternative methods could be important, at least as a first step in the hazard assessment of chemicals and environmental samples (Scholz et al. 2013). For instance, the use of in vitro bioassays that can be applied to water, sediment, and soil samples (that could be applied in combination with analyses of organisms and fish tissues captured in the wild) constitutes a feasible and valuable tool that can be easily implemented (Quesada‐García et al. 2015).

### How effective are protected areas, including terrestrial (e.g., parks, wildlife corridors), freshwater, and marine habitats, to safeguard biodiversity from the impact of environmental pollutants?

In the current biodiversity crisis (Vörösmarty et al. 2010; Loehle and Eschenbach 2012), the creation of protected areas (PAs) represents an essential approach to the conservation of biodiversity and ecosystems (Dudley 2008). In recent years, the number of PAs has significantly increased in LA countries in the pursuit of biodiversity protection (Naughton‐Treves et al. 2005). Nevertheless, despite evidence demonstrating that pollution adversely affects populations (Oehlmann et al. 1996; Guillette et al. 1999; Willemsen and Hailey 2001; Blaustein and Kiesecker 2002; Kidd et al. 2007; van de Merwe et al. 2010), the extent of the impact of pollution inside the PA system has not been a main topic either in the scientific literature or within the PA manager's work environment (Rodríguez‐Jorquera et al. 2016, 2017). For instance, Frazier (1999) determined that for Ramsar Convention wetlands located in the Neotropical ecozone (including all LA countries), pollution was the most commonly reported factor of change. Furthermore, in an analysis of the extent of pollutant occurrence inside LA PAs, Rodríguez‐Jorquera and collaborators (2017) found 119 cases of chemical pollution occurrence inside PAs in 16 LA countries. Among these cases, mining and hydrocarbon extraction were the main sources of pollution, and aquatic environments appear to be the most threatened habitats (Rodríguez‐Jorquera et al. 2017). In all these cases, research to determine the generally invisible effects of pollutants on the affected ecosystems was virtually absent, and when present, reactive to major and evident ecosystem damage (Rodríguez‐Jorquera et al. 2017). The effectiveness of PAs in protecting biodiversity from pollution effects remains a critically important concern because PAs are not environmentally isolated. For example, PAs close to anthropogenic activities have experienced chemical exposure and associated adverse biological effects (Araujo et al. 2013). Contamination control at the source represents the most logical step to manage adverse effects of pollution inside LA PAs. Comprehensive ecotoxicological research, including the integration of pollutants determination and their effects on biota, and building technical capacities (human and instrumental) are emerging as necessary first steps to establish monitoring priorities. If advanced, these first steps could also serve to improve policies and regulations to safeguard the biodiversity of LA PAs.

### How can we improve risk management approaches for solid wastes (e.g., landfill leachate, incineration) in Latin America?

More than half (54%) of the total LA and Caribbean municipal wastes are deposited in landfills (ONU‐HABITAT 2012). Unfortunately, solid waste management practices and treatment technologies vary dramatically among regions of LA, resulting in differential protection of the environment and human health from risks posed by contaminants of historical and emerging concern. Landfill leachates contain diverse chemicals, which are introduced to groundwaters and surface waters. Potential chemical exposures to human populations and ecosystems resulting from these leachates are particularly important in many parts of LA because most landfills are unlined and landfill leachates are untreated. For example, unused antibiotics are often discarded to landfills and are found in leachates (Holm et al. 1995; Lu et al. 2016). In fact, antibiotic occurrence in the environment influences the development of antibiotic resistance, which now represents a leading global threat to public health (http://www.who.int/antimicrobial-resistance/en/). For example, influences of antibiotics in the environment on the development of antibiotic‐resistant microorganisms was recently identified as a priority research need by an expert workgroup (Boxall et al. 2012). Research is needed to understand chemical contaminants and ecotoxicity associated with leachates, to comparatively examine treatment efficiencies of technologies in various regions, and to identify optimal management alternatives for LA.

## SPOTLIGHT ON LATIN AMERICA

### What is the sensitivity of regional species to contaminants that will allow us to better predict impacts on local ecosystems?

Environmental hazard and risk assessments that employ probabilistic species sensitivity distributions (SSD) represent the most widely used approach by regulatory entities worldwide to derive acceptable environmental concentration limits for protection of structure and function of ecosystems (Zajdlik et al. 2009; Dowse et al. 2013; Wang et al. 2015). The theoretical basis of SSD is that it is possible to describe the variability and range of sensitivities among individual taxa across trophic positions with a statistical or empirical function (Posthuma et al. 2002). However, there are still issues to be resolved with the SSD method, one of them being the selection of species to be included. Ideally, all species in a specific ecosystem should be considered, and the data set should be statistically and ecologically representative of the ecosystem (Wang et al. 2015). This aspect is often problematic in LA because toxicity data for endemic indigenous or native species are habitually lacking. Usually LA species are within the most sensitive species of the world (Carriquiriborde and Ronco 2002). It is therefore important to promptly develop and implement LA‐specific ecotoxicity studies, testing, and innovative field assessment protocols (e.g., environmental DNA barcoding) (Brodeur and Vera Candioti 2017; Xie et al. 2017) using native species. Such efforts will contribute to better understanding of known native species, and to sensitivity differences compared with those species commonly used in other regions (e.g., Northern Hemisphere). In turn, these advances will help to better predict impacts on local ecosystems and delineate meaningful environmental quality criteria.

### Which laboratory studies can we develop to contribute to the risk assessment of natural resource extraction (oil, gas, mining) in Latin American ecosystems?

Latin America holds 40% of the world's biological diversity, 30% of Earth's available freshwater, and almost 50% of the world's tropical forest). At the same time, it is the world's leading source of metals and the second most important source of oil (ECLAC 2013). The region produces 15% of the world's Au, 45% of Ag, and 40% of Cu, and holds almost 20% of the global proven reserves of oil and 40% of the total reserves of unconventional oil and gas (EIA 2015). In this context, a clear challenge for the region therefore consists in being able to manage such natural resource extractions without polluting and disrupting the rich and varied biodiversity and ecosystems of LA. The potential environmental impacts from oil, gas, and mining projects are numerous, including negative impacts on air, land, and water quality; greenhouse gas emissions; oil spills; and effluent discharges. During exploration, seismic lines can disturb significant amounts of vegetation, and during production, there can be a considerable amount of dredging and filling of the waterways, leading to acidification of water bodies, erosion, and spills (Ingelson and Nwapi 2014). In addition, accidents during operations (e.g., oil spills) can be catastrophic in high‐diversity ecosystems. In recent years, the social and environmental impact of accessing nonconventional gas reserves through the technique of hydraulic fracturing (or “fracking”) has also emerged as an issue of concern around the world, including LA. The technique relies on the use of fluids consisting of a mixture of water, sand, chemicals, and additives (e.g., viscosifiers, surfactants, pH control agents, biocides) that are combined and injected into the coal seam at high pressure to aid the fracturing process. Benzene, toluene, ethylbenzene, and xylene (jointly referred to as “BTEX”) can also be released during the fracking process (Burton et al. 2014). Uncertainties remain regarding the health and environmental safety of fracking chemicals, and concerns exist that these and the BTEX may disperse into the groundwater aquifers or surface waters or volatilize into air (Colborn et al. 2011; Burton et al. 2014). Research is needed to define environmental quality criteria and management strategies associated with conventional and unconventional natural resource extraction that specifically accounts for the biodiversity and unique ecosystems in LA. As noted above, it will be necessary to include approaches using local species relevant to regional ecological protection goals.

### How can we develop, validate, and apply ecotoxicological tools useful for characterizing and classifying industrial and residential effluents in Latin America?

With 75% of its inhabitants living in cities, LA is the region of the world where the greatest proportion of the population lives in urban centers (Bárcena 2001). As such urbanization occurs, concentration of chemical use and access to chemical products are increasing faster than environmental management systems can be implemented in many regions (Corrales et al. 2015). In spite of such pronounced urbanization, implementation of wastewater treatment systems for residential effluents still lags considerably behind developed countries, with less than 10% of domestic sewage being treated by water reclamation plants (UNEP 2002). This situation is further complicated by the lack of reliable information on industrial effluent discharges, which are often released within residential sewage collection systems. As previously introduced, national measures of industrial emissions are lacking in LA (Jenkins 2000), a fact that illustrates the widespread absence of control by governments, as they appear unwilling or unable to enforce environmental regulations on industrial producers (US Department of Commerce 2002). The lack of adequate effluent treatment is a major environmental problem that needs urgent solutions as untreated sewage and effluents enter rivers, lakes, underground aquifers, and oceans. Food safety concerns inherently arise when such waste streams of differential quality are reused for terrestrial agriculture and aquaculture. To facilitate control and promote reduction of pathogen and chemical contamination and associated risks to human populations and ecosystems, efforts should be invested in implementing waste reduction technologies, including green chemistry and engineering, while developing, validating, and implementing whole effluent toxicity (WET; Grothe et al. 1996) and ambient testing strategies and methods (La Point and Waller 2000) adapted to LA species and ecosystems (USEPA 2002; Bundschuh 2014).

## CONCLUSIONS

The pursuit of sustainable environmental quality to protect public health and the environment is a compulsory long‐term goal shared by most modern societies and civilizations worldwide. In the present study, we report findings of an innovative initiative aimed at identifying key priority environmental research needs in the fields of environmental toxicology and chemistry, which are necessary to achieve more sustainable environmental quality in LA. One hundred research questions were initially received from environmental experts in the academic, government, and industry sectors from numerous LA countries. Twenty priority research questions were identified during the LA GHSP workshop in Buenos Aires and organized in 6 categories: Risk Assessment; Environmental Chemistry; Ecotoxicology; Health, Contaminants of Emerging Concern, and Environment; Spotlight on LA; and Environmental Management and Policy. This exercise also identified limited communication and coordination among governmental, industrial, and academic sectors in LA. We hope this horizon scanning effort will provide a foundation from which future bridges can be built among these sectors to facilitate strategic research activities and implementation of policies that are more environmentally sound.

Identified issues of concern included the development, improvement, and harmonization across LA countries of methods for 1) measuring concentrations of contaminants and degradation products in complex matrices (i.e., biota); 2) better predicting effects of contaminants on ecosystems, addressing lab‐to‐field extrapolation problems and the complexity of mixtures or multiple stressors (including climate change); and 3) estimating environmental risk (i.e., risk characterization of mixtures of diffuse contaminants) and improving management and regulatory tools (i.e., maximum limits of toxicity) toward achieving sustainable development. In addition, those environmental contaminants frequently addressed in the questions were pesticides and emerging pollutants (pharmaceuticals, EDCs, plastics, nanomaterials). Major and consistently identified environmental issues were related to agriculture (mainly pesticides), industrial and urban effluents, solid wastes, pulp and paper mills, and extractive activities (oil, gas, and mining). Several special topics of concern included assessing and preventing pollution impacts on wildlife protected areas, developing strategies for identification, substituting and designing less hazardous chemicals for human health and the environment, and the difficulty of establishing and implementing allowable limits for emerging contaminants in environmental legislation across LA. Finally, a recurrent research need included gaining more information and a better understanding of differential sensitivities among regional species and ecosystems to environmental contaminants and other stressors.

The effort described in the present study is part of a larger effort to identify important international research needs to advance more sustainable global environmental quality (Brooks et al. 2013). This transparent, inclusive, multidisciplinary, and bottom–up process is already supporting strategic research planning and engagement across disciplines and among countries. For example, priority research needs are being integrated within special symposia, focused‐topic workshops, and themes of scholarly meetings in various parts of the world. We hope the priority research needs identified in the present work will be useful during the development and implementation of strategic environmental quality research programs in LA.

## Disclaimer

The authors declare no conflicts of interest.

## Data Accessibility

This article has no metadata and calculation tools. All the raw data can be found in Table S1 in the Supplemental Data.

## SUPPLEMENTAL DATA

**Table S1.** Questions submitted from Latin American scientists and engineers and examined during a synthesis workshop in Buenos Aires, Argentina

## Supporting information

This article includes online‐only Supplemental Data.

**Table S1.** Questions submitted from Latin American scientists and engineers and examined during a synthesis workshop in Buenos Aires, Argentina.
