# Supplementary material for: Toward sustainable environmental quality: Identifying priority research questions for Latin America
Source: Integr Environ Assess Manag. 2018 Feb 22;14(3):344–57. doi: 10.1002/ieam.2023 (PMC5947661; doi:10.1002/ieam.2023)
Supplement: Supplementary file 1 — Table S1. Questions submitted from Latin American scientists and engineers and examined during a synthesis workshop in Buenos Aires, Argentina. [file IEAM-14-344-s001.docx]

**SUPPLEMENTARY INFORMATION**

**Towards Sustainable Environmental Quality: Priority Research Questions for Latin America**

Furley TH^1^, Brodeur JC^2^, Silva de Assis HC^3^, Carriquiriborde P^4^, Chagas KR^1^, Corrales J^5^, Denadai M^6^, Fuchs J^7^, Mascarenhas R^8^, Miglioranza KSB^9^, Miguez Caramés DM^10^, Navas, JM^11^, Nugegoda D^12^, Planes E^13^, Rodriguez-Jorquera I^14^, Medina MO^15^, Boxall ABA^16^, Rudd MA^17^, Brooks BW^5^

^1^ Aplysia Environmental Consulting, Vitória, ES, 29090-210, Brazil

^2^ Instituto de Recursos Biológicos, Centro de Investigaciones de Recursos Naturales (CIRN), Instituto Nacional de Tecnología Agropecuaria (INTA), Hurlingham, Buenos Aires, Argentina

^3^ Pharmacology Department, Federal University of Parana, Curitiba, Brazil

^4^ Centro de Investigaciones del Medio Ambiente, Facultad de Ciencias Exactas, Universidad Nacional de la Plata – CONICET. Calle 115 s/n, La Plata (1900), Buenos Aires, Argentina

^5^ Department of Environmental Science, Baylor University, Waco, Texas, USA

^6^ Department of Chemistry, Federal University of São Carlos, São Carlos, Brazil

^7^ IQUIBICEN-CONICET, Toxicología y Química Legal, Departamento de Química Biológica, Facultad de Ciencias Exactas y Naturales, Universidad de Buenos Aires, Int. Güiraldes 2160 (C1428EHA), Ciudad de Buenos Aires, República, Argentina

^8^ Universidade Estadual de Feira de Santana, Feira de Santana, Brazil

^9^ Lab. Ecotoxicología y Contaminación Ambiental, IIMyC, CONICET- UNMDP, Argentina.

^10^ Laboratorio Tecnológico del Uruguay (LATU), Montevideo, Uruguay

^11^ INIA, Department of Environment, Ctra. De la Coruña Km 7.5, E-28040 Madrid, Spain

^12^ RMIT University, Melbourne VIC 3001, Australia

^13^ National Institute of Industrial technology, Chemistry Center, 1650 San Martin, Provincia de Buenos Aires, Argentina

^14^ Centro de Humedales Río Cruces, Universidad Austral de Chile, Las Encinas 220, Anexo Ex Hotel Isla Teja, 4to Piso, Pabellón B, Valdivia, Chile

^15^ University of Guadalajara, Guadalajara, Mexico

^16^ Environment Department, University of York, Heslington, York, YO10 5NG, UK

^17^ Department of Environmental Sciences, Emory University, Atlanta GA 30322, USA

**Supplementary Table 1.** Questions submitted from Latin American scientists and engineers and examined during a synthesis workshop in Buenos Aires, Argentina.

| **1** | What is the real impact of agrochemical diffuse pollution on agricultural ecosystems and their functions? Is the current level of agrochemical use in extensive farming sustainable? |
| --- | --- |
| **2** | Do exist worldwide laws that consider the risk of the simultaneous use of two or more agrochemicals? Have chronic toxicity-tests been proposed, to legislate on the use of agrochemicals? |
| **3** | What are the risk factors that must necessarily be evaluated in order to respond in full awareness on the environmental sustainability, or not, of the "new" technologies of oil and gas extraction? |
| **4** | Given the difficulty of extrapolating results obtained in the laboratory to field conditions ... What is the degree of certainty we can give to conclusions from studies conducted under laboratory conditions? |
| **5** | What is the rate of change in both physicochemical and biological parameters, in a natural environment without the direct effect of human activities? |
| **6** | Will it be sufficient to continue using the classical physicochemical and bacteriological determinations to assess the quality of water and soils, without incorporating ecotoxicological assessment? |
| **7** | What is the effect of the use of biosolids in agriculture, fruit growing and livestock on groundwater? How should the risks assessment on human health and primary products be performed? |
| **8** | Are the environmental risk assessment valid tools for evaluating sustainable use of chemicals? |
| **9** | How should the evaluation of environmental risks of pesticide use in eucalyptus plantations for the paper industry be made? |
| **10** | Pesticides can be beneficial and not harmful to the fish in their natural environment? |
| **11** | What water quality parameters are critical for determining the abundance and diversity of fish in aquatic ecosystems? |
| **12** | What compounds could replace the use of pesticides? |
| **13** | What are the environmental levels of major sold and used agrochemicals in the country? Are needed frequent and reliable surveys to determine the degree of exposure? |
| **14** | What are the levels of pollution by plastic waste? microplastics? or toxic compounds adsorbed on the surface of the plastic? |
| **15** | What organic compounds could be used as markers of different sources of pollution in aquatic environments? |
| **16** | Considering the bioaccumulative potential of some active pharmaceutical compounds on aquatic organisms, how to assess the implications in a long-term perspective for humans? |
| **17** | What are the main drugs and their metabolic by-products that can be found in aquatic environments, what are their ecological effects and how the environmental legislation in Latin American countries may be appropriate to restrict the reach of potential environmental impacts? |
| **18** | Which groups are currently working with the monitoring of toxic substances such as TBT used in paint? It is possible to standardize more species that could indicate sensitivity when in contact with toxic substances? |
| **19** | How can SETAC encourage researchers from universities and institutes to develop more studies/tools in environmental forensics? |
| **20** | Are there any recent analyses to detect the presence of PAHs in biological organisms, specifically with plants that could be used in environmental monitoring of urban/industrial areas? |
| **21** | There is a validated method for glyphosate determination in animal tissue? |
| **22** | What it happen with pesticides mixtures at the environmental level? How does it behave? Is it persistence, mechanism of action and other characteristics modified with the time? |
| **23** | Are emerging pollutants of regional importance toxic for aquatic and terrestrial biota associated with the aquatic environment? |
| **24** | Are "model" testing species representative to determine the potential adverse effects of pesticides on aquatic biota in Latin America? |
| **25** | How are rain forest ecosystems, in particular freshwater ecosystems, being affected by oil and chemical spills caused by the oil industry? |
| **26** | How should it be done to include a highly sensitive local species in monitoring program of my country? How should it be done to include it in worldwide standardize tests? |
| **27** | Have native species similar biological responses than standard species when they are exposed to environmental pollutants of regional importance? |
| **28** | What will it be the environmental liabilities related with the latest technologies in oil and gas extraction? |
| **29** | Up to which point the use of native fish is a good tool for assessing the quality of water resources, considering their chronic exposure to environmental contaminants and their adaptation mechanisms to that environment? |
| **30** | Which would be the marine fish species that could be standardized and used for evaluation studies of industrial activities and for the oil exploitation activities (such as explosions)? |
| **31** | What are the impacts of iron, manganese and copper mining in tropical countries? What should be the evaluation protocol to be followed? |
| **32** | What is the ecotoxicity status of domestic and industrial effluents (per sector) of Latin America, what are the main causes of toxicity and what to do to reduce it? |
| **33** | Trials to evaluate the toxic potential of chemicals should not also be carried out with the water in the area in which they intend to use the product and use as test organism endemic species ? |
| **34** | the ecotoxicological assessment of industrial effluents should not cover beyond the traditional assays, with endemic species as well? seeking greater proximity to the result to be expected in the environment. |
| **35** | What is the real knowledge we have about the effects of pollution at Latin America scale? Is it possible a good methodological approach on the bases of the current evaluation requirements for the scientific system? |
| **36** | perform a Latin American protocol for ecotoxicological control gear and industrial and agricultural effluents , since entering the protocol recommended limits as well as step to investigate the cause ( physical and / or chemical ) of the environmental effects |
| **37** | as it should be a ecotoxicological control protocol for industrial waste deposited in soils in Latin ? |
| **38** | as it should be conducted environmental control, and what are the steps to reduce the impact (if identified) of coffee plantations in tropical countries cane sugar ? |
| **39** | What are the endpoint thresholds for each compound on the different wild species commonly exposed to pesticides and on humans? and what are the possible short and long term effects? |
| **40** | How do concentrations of majoritarian ions affect the aquatic biota in each region of the planet? |
| **41** | Are the glyphosate and/or its commercial formulations endocrine disruptors? |
| **42** | Could climate change (pH, temperature) affect the mechanisms of action of contaminants as we know them? |
| **43** | Are there effects of climate change on environmental stress factors? In turn, could these changes affect environmental sustainability? |
| **44** | Does it exist a common factor that explain the toxic action of a contaminant on individuals and the effects induced at ecological levels? |
| **45** | How can laboratory in vitro approaches be used to characterize oil pollution sources and effects on rain forest water and sediment systems? |
| **46** | How to predict the toxicity of a contaminant in sediments with very different natures? |
| **47** | It will be possible to add sublethal toxicity testing for assessing exposure to pollutants and toxic compounds for the risk management? |
| **48** | What is the resilience time a population exposed to pesticides required for showing non-cumulative changes? |
| **49** | How the potential of omics technologies should be exploit for assessing the action of toxic contaminants? |
| **50** | Is it possible to generalize the results of regulatory single species toxicology test on a complete taxon species in different ecosystems, climates or physicochemical conditions? |
| **51** | How is it that certain species of live fish, or even abound in areas surrounding raw sewage discharges? |
| **52** | Observational fieldwork is rarely addressed in ecotoxicological studies of pesticides, why? Therefore, I would like to know why the importance of fieldwork has not been recognized within the ecotoxicology. |
| **53** | The ecotoxicology study of neotropical amphibians. The study of the interaction between climate change and chemical contamination. The study of the interaction between multiple environmental stressors. |
| **54** | How to scale the intensity of environmental damage at different levels of biological organization and use this information in consistent programs evaluation and environmental monitoring? |
| **55** | How to meet the need of new ecotoxicological tests from tests already existent as standards? |
| **56** | Is it enough for regulation the use of toxicology test based only on active ingredients and not commercial formulations of pesticides? |
| **57** | what are the estuarine aquatic species to be used in chronic tests with water and sediment ? |
| **58** | What important/How strong are the risks that produce pharmaceuticals in the environment? What should we do to respond to this question? And should we do it in conjunction with the pharmaceutical industry? |
| **59** | What is the real magnitude of the impact of drugs in the environment and biota? |
| **60** | Are blue-green algae blooms direct or indirect threat to fish? |
| **61** | What are the main hormone disrupters, how they are introduced to the environment and what are the ecological consequences of their release in natural aquatic environments such as rivers and lakes in Latin America? |
| **62** | What would be the impact of micro-plastics in the physiology of marine organisms? |
| **63** | Which are the environmental variables (abiotic factors) that trigger the production of toxins by cyanobacteria in the environment? |
| **64** | What are the real impacts of nanomaterials on the aquatic environment, on animals that live in it, on human’s health and on monoclonal cells and 3D cultivation? |
| **65** | For substances like pesticides of pharmaceuticals, are the mechanisms of action which they were designed for or different ones those that should be studied to observe potential adverse effects on non-target organisms? |
| **66** | Does the presence of contaminants in regional ecosystems significantly impacts human food security? |
| **67** | Does the presence of contaminants in regional ecosystems significantly impacts human health? |
| **68** | How are the effects of atmospheric contaminants in plants related to effects upon humans? |
| **69** | How to relate the results of ecotoxicological from domestic effluents, with the quality of water for human consumption and the reduction of costs with drinking water treatment? |
| **70** | aligning the reduction of environmental effects of organic agricultural crops as the benefits to human health ? |
| **71** | What are those pesticides applied on food for human consumption that should withdraw from the market, because they are uptake by the product and are consumed by humans, being potentially bioaccumulated and transferred to the offspring, causing diseases and hormonal disorders? |
| **72** | What are the relationships between inputs to human society and environmental health risks? What are the limits of acceptance of the risks? |
| **73** | What would be the progressive goals of continuous improvement to the maximum limits of toxicity allowed from domestic and industrial effluents? |
| **74** | How to economically demonstrate to industries the possibility to achieve a reduction in effluent toxicity within the production process reducing costs in environmental monitoring and costs of the use of chemicals in the process? |
| **75** | How to account for environmental damage, for penalty purposes, from ecotoxicological tests’ results? |
| **76** | How to make ecotoxicology sciences really reach employees of environmental agencies of at least all the states and provinces capitals of the Latin American countries? |
| **77** | How to make executive power posts in the ministries related to environmental issues are occupied by qualified technicians for the job? |
| **78** | how would the improvement progressive goals remains plausible for Latin americandos countries of reduction of the potential environmental effects of pesticides and other chemical agricultural products , products used in industry and household usage |
| **79** | Is a large-scale agriculture possible without the use of pesticides? |
| **80** | Why environmental protection laws are not met in Latin America? Are these laws are insufficient? |
| **81** | How effective are protected areas to safeguard biodiversity from the impact of environmental pollutants? |
| **82** | What actions should be followed by all countries to standardize and harmonize the care of ecosystems through the use of ecotoxicological methodologies in environmental management? |
| **83** | Is biotechnology a solution to the problems of soil contamination by persistent organic pollutants? |
| **84** | How emissions and waste disposal are assessed in the various countries of Latin America? |
| **85** | Whereas part of the agricultural production in Latin America, such as soybeans, is intended for biofuels production, are we working on any predictive model for estimating the environmental cost, oriented to define policies for clean energy technology development? |
| **86** | What are the regulatory frameworks that were most effective for improving environmental management of chemicals and chemical products? How the effectiveness of regulatory actions can be evaluated? |
| **87** | Is it being developed locally some regulation to control the use and animal welfare, considering all taxa used in experiments? |
| **88** | In which way could toxicologists move/persuade governmental agencies to modify existing laws to accept the results of the research focused on environmental toxicology? |
| **89** | How the concept of "ecosystem services" could be incorporated into national law? How these laws could be more severe for penalizing companies or people that alter and harm the environment? |
| **90** | Do national or provincial laws exist to regulate the simultaneous use of two or more agrochemicals? Does it take into account the single or combined chronic effects? |
| **91** | What alternatives can be developed to improve the leachate from landfills? |
| **92** | Is it being developed locally some regulation to control the use and animal welfare, considering all taxa used in experiments? |
| **93** | Is it enough for regulation the use of toxicology test based only on active ingredients and not commercial formulations of pesticides? |
| **94** | To reduce the gap between scientific knowledge generated empirically and its application for human benefit. |
| **95** | Which factors can justify the lack of research and dissemination of terrestrial ecotoxicology? |
| **96** | I believe in research of both inter and multidisciplinary nature. The creation of research funds to researchers with different methodological tools to be able to study the same problem more broadly. |
| **97** | How can one persuade politicians about the need to increase investments in environmental research? |
| **98** | How academia (universities) could become advisors of government agencies related to environmental protection? |
| **99** | Regulatory toxicological test for new chemicals: should it be left to the companies that produce such products? |
| **100** | How to broaden toxicology teaching at different levels of education, from primary school, undergraduate courses in different areas and post-graduation degrees? |
